# Analysing 3429 digital supervisory interactions between Community Health Workers in Uganda and Kenya: the development, testing and validation of an open access predictive machine learning web app

**DOI:** 10.1186/s12960-021-00699-5

**Published:** 2022-03-16

**Authors:** James O’Donovan, Ken Kahn, MacKenzie MacRae, Allan Saul Namanda, Rebecca Hamala, Ken Kabali, Anne Geniets, Alice Lakati, Simon M. Mbae, Niall Winters

**Affiliations:** 1 Division of Research and Health Equity, Omni Med Uganda, Mukono District, Makata, Uganda; 2 Learning and New Technologies Research Group, Department of Education, University of Oxford, Oxford, United Kingdom; 3 Department of Medicine, Tufts University School of Medicine, Boston, United States of America; 4 Division of Health and Social Sciences, Amref International University, Nairobi, Kenya; 5 Division of Research, Medic Mobile, Nairobi, Kenya

**Keywords:** Machine learning, Artificial intelligence, Supervision, Community Health Worker, Digital Health, Training

## Abstract

**Background:**

Despite the growth in mobile technologies (mHealth) to support Community Health Worker (CHW) supervision, the nature of mHealth-facilitated supervision remains underexplored. One strategy to support supervision at scale could be artificial intelligence (AI) modalities, including machine learning. We developed an open access, machine learning web application (CHWsupervisor) to predictively code instant messages exchanged between CHWs based on supervisory interaction codes. We document the development and validation of the web app and report its predictive accuracy.

**Methods:**

CHWsupervisor was developed using 2187 instant messages exchanged between CHWs and their supervisors in Uganda. The app was then validated on 1242 instant messages from a separate digital CHW supervisory network in Kenya. All messages from the training and validation data sets were manually coded by two independent human coders. The predictive performance of CHWsupervisor was determined by comparing the primary supervisory codes assigned by the web app, against those assigned by the human coders and calculating observed percentage agreement and Cohen’s kappa coefficients.

**Results:**

Human inter-coder reliability for the primary supervisory category of messages across the training and validation datasets was ‘substantial’ to ‘almost perfect’, as suggested by observed percentage agreements of 88–95% and Cohen’s kappa values of 0.7–0.91. In comparison to the human coders, the predictive accuracy of the CHWsupervisor web app was ‘moderate’, suggested by observed percentage agreements of 73–78% and Cohen’s kappa values of 0.51–0.56.

**Conclusions:**

Augmenting human coding is challenging because of the complexity of supervisory exchanges, which often require nuanced interpretation. A realistic understanding of the potential of machine learning approaches should be kept in mind by practitioners, as although they hold promise, supportive supervision still requires a level of human expertise. Scaling-up digital CHW supervision may therefore prove challenging.

*Trial registration*: This was not a clinical trial and was therefore not registered as such.

**Supplementary Information:**

The online version contains supplementary material available at 10.1186/s12960-021-00699-5.

## Background

By 2030 The World Health Organization (WHO) estimates there will be a global shortage of 18 million health workers, which will be most pronounced in countries defined as low- or middle-income (LMIC) [1]. To address this gap in human resources for health, Community Health Workers (CHWs) have been trained to deliver primary healthcare services [2], especially in remote or rural communities.

Although there is no fixed definition for a CHW, the term is generally used as an umbrella description for groups of “…paraprofessionals or lay individuals with an in-depth understanding of the community, culture and language, who have received standardised job-related training of a shorter duration than health professionals and whose primary goal is to provide culturally appropriate health services to the community” [3].

In 2010, a report from the WHO stated that supervision is one of the “weakest links in CHW program(me)s” [4], for reasons including a shortage of supervisors and poorly designed programmes. As a result, the use of mobile technologies (mHealth) has been proposed as one way to address these challenges [5, 6] and in 2018 a $100 million fund was announced at The World Economic Forum to support mHealth-facilitated training and supervision of 50,000 CHWs across sub-Saharan Africa [7]. Yet, from a pedagogical perspective, the evidence regarding the use of mHealth to support CHW supervision is weak. A systematic scoping review by Winters et al. found that of 24 studies which described the use of mHealth to support CHW training and learning, only four drew upon established theories of learning [8]. The authors of this study suggest that “mHealth suffers from a reductionist view of learning that underestimates the complexities of the relationship between pedagogy and technology” [8]. It is therefore vitally important that we understand the nature of mHealth-facilitated supervision occurring between CHWs in order to ensure it facilitates CHW learning and professional development in a rigorous manner.

To try and capitalise on the promise of mHealth to support CHW supervision, interactive forms of learning—which are supported by the technological capabilities of mobile technologies—are beginning to be explored [9]. Examples include the use of instant messaging applications (apps) to encourage interactive and peer-to-peer forms of learning [10]. Such approaches could help to facilitate a move away from less pedagogically sophisticated means of supervision, which have traditionally focussed on simplistic, information dissemination style interventions (e.g. one way messaging) [8, 11, 12]. These have been critiqued in the wider literature for simplified approaches to supervision which fail to promote CHW collaboration, agency and professional growth [8, 12].

Yet, despite emerging attempts to understand how the use of instant messaging apps can support CHW supervision [13, 14], the analysis and processing of message exchanges remains a time-consuming and labour intensive task. The small number of existing studies on this topic have relied on manual coding of messages to understand the nature of supervisory interactions [13, 14]. Although this is feasible for small scale pilot studies, the rapid growth of large-scale CHW programmes (many of which involve the supervision of thousands of CHWs) [15] means that alternative strategies need to be explored to allow CHW programme managers to better support CHW supervision at scale.

One such strategy could be through the use of machine learning. Machine learning is a sub-field of artificial intelligence (AI) where computers “learn from a set of data and subsequently make predictions” [16]. The use of machine learning has been explored in other areas of healthcare, such as automated processing of radiological imaging and prediction of ocular pathology [17, 18], however, as of yet, its role in supporting CHW supervision has not yet been explored.

## Methods

### Study aims

This study aims to document the development, testing and validation of an open access machine learning web app (CHWsupervisor) to understand whether it can accurately analyse CHW messaging exchanges compared to human coders, and to determine the potential for its use at scale. We also aim to explore the nature of supervisory messages exchanged between CHWs and CHW supervisors, as well as the time taken by the machine learning web app to code datasets of messages compared to human coders. Finally, we aim to document the challenges of adopting a machine learning approach to analyse supervisory exchanges.

### Study design

This is a descriptive development and validation study carried out between March 2020 and April 2021.

### Data sets used for web app development and validation

The CHWsupervisor web app was developed and validated using messaging data exchanged between CHWs on WhatsApp [19]. WhatsApp is a publicly available instant messaging mobile App and free to download. It is the most popular mobile messaging App in sub-Saharan Africa, with an estimated 1.5 billion users globally [20]. A key feature of WhatsApp is that it allows users to create and participate in group chats. It also allows users to record and send voice-notes, which is especially beneficial to illiterate users.

The CHWsupervisor web app was trained using messages obtained from a WhatsApp network in Uganda, involving CHWs (*n* = 12, CHW peer-supervisors (*n* = 2), healthcare workers (*n* = 2), and research project facilitators (*n* = 3). Throughout the manuscript, this dataset is referred to as the ‘training dataset’. Messages in this training data set were exchanged between January and July 2019 and came from two different WhatsApp groups—Group A and Group B:

- WhatsApp Group A contained 1109 messages and was primarily used to share logistical day-to-day information between CHWs and their supervisors (e.g. coordinating meetings and informing CHWs of training sessions) sending greetings, and the discussion of any other miscellaneous tasks.
- WhatsApp Group B contained 1078 messages and was designed as a space for both formal and informal learning. Here CHWs could engage with structured clinical cases released on a bi-monthly basis (which were moderated by the CHW facilitators), as well as share their own informal cases which they had encountered during their daily practice.

The CHWsupervisor web app was then validated on 1242 messages obtained from a WhatsApp network in Kenya, which involved CHWs (*n* = 25), CHW supervisors (*n* = 8), NGO officials (*n* = 3), Ministry of Health officials (*n* = 2), research project facilitators (*n* = 2), and a Community Health Committee representative (*n* = 1). Messages from this data set were exchanged between August 2014 and March 2015. Throughout the manuscript, this dataset is referred to as the ‘validation dataset’.

### Data analysis

i. Sorting and organising of messages
  WhatsApp messages from all datasets were downloaded to a Microsoft Excel spreadsheet and sorted based on the date and time they were sent using the automated ‘Sort and Filter’ function in Microsoft Excel. Each message was read in turn by one member of the research team. Personal identifiers were removed from messages to preserve anonymity. Text messages sent in Luganda or Swahili were translated to English, and voice note messages were translated and transcribed into English. The translation was done by two members of the research team who were fluent in English and Luganda or Swahili, respectively. This was then double checked by two other members of the research team who were also bilingual to ensure consistency. Where there were disagreements about precise translation, a discussion was held and a final translation was decided on. Blank, non-sensical, media, and duplicate messages were removed from the datasets prior to analysis (for further information on removed messages please refer to the Additional file 1).
ii. Manual coding of messages
  Individual messages across all WhatsApp datasets were coded based on the perceived supervisory objective of the message. This was done using a deductive approach by drawing upon an existing framework published by Vu Henry et al. [13], which was based on initial work undertaken by Perry and Crigler to investigate how supportive supervision of CHWs contributes to health systems strengthening [21].

This framework contains three main categories of supervision:

1. Communication and Information (e.g. messages pertaining to clarifying, giving or requesting information; exchanging logistical information; acknowledgements). Such examples might include:
  “Please could you tell us where we are going to meet today to visit patients?”
  “We need to complete the patient data forms to be submitted to the health centre this week—please remind your colleagues.”
2. Quality Assurance (e.g. messages regarding consent taking; health education; follow-up or outreach work; record keeping; safe patient management). Such examples might include:
  “Please ensure the consent forms are signed by the patients you visit.”
  “John*, the patient you saw with the discharging ear will require antibiotic ear drops and follow-up, as it is likely they have Chronic Suppurative Otitis Media.”
3. Supportive Environment (e.g. messages focussed on encouragement or praise; sending greetings; providing moral support giving thanks or inspiration; sending an apology). Such examples might include:
  “Well done to everyone who attended today! You did a wonderful job.”
  “Keep up the great work in the community.”

Each dataset was read individually by one member of the research team and assigned a code based on the above supervisory objectives. This was done in a hierarchical fashion, since sometimes messages contained multiple supervisory objectives. This meant messages were assigned primary, secondary and/or tertiary codes where necessary. The same individual then repeated this process two months later to check for any discrepancies. Where necessary, modifications were made and messages were re-coded. This process took ~ 6 weeks. A second member of the research team then coded the data sets. Following this process both coders met virtually via Skype over a period of 4 weeks to discuss each message in turn (usually 75–100 messages in one sitting) and came to a unified agreement as to the final coding system. This process was done consecutively.

### CHWsupervisor web app design and development

The CHWsupervisor web app was developed over a 9-month period (between March and November 2020) and uses the open access Tensorflow.js [22]; a JavaScript deep machine learning library, developed by Google. This library was first used to encode all 2187 messages from Groups A and B using the Universal Sentence Encoder [23]; a deep learning model trained to produce a vector of 512 numbers for any English sentence. The Universal Sentence Encoder model was trained to produce encodings that were instrumental in good performance on a variety of natural language tasks. The CHWsupervisor web app was trained with encodings from the Universal Sentence Encoder and an optional encoding of the role of the message senders. It was trained to match the message category labels provided by the human coders. CHWsupervisor can also be used by groups who wish to categorise messages differently from how it was done in this study. They need at least two spreadsheets of messages, one of which has been annotated with category labels. The app can then label the remaining unlabelled spreadsheets.

It is common practice in machine learning to adjust the parameters that define the machine model's architecture and training settings. A period of fine-tuning was therefore undertaken to find the best parameters for the web app.

To take advantage of a higher-level interface compared to TensorFlow [24], CHWsupervisor was implemented in *Snap!* [25]. The web app and source code are freely available at: https://ecraft2learn.github.io/ai/snap/snap-no-logging.html?project=CHWsupervisor. For the libraries underlying CHWsupervisor and further specific technical details on the development process, please refer to the Additional files 2 and 3.

To validate CHWsupervisor, we trialled it on a validation data set containing 1242 instant messages exchanged between CHWs and supervisors in Kenya.

For both the training (Uganda) and validation (Kenya) datasets, we compared the suggested codes generated by the CHWsupervisor web app with a set of combined codes that were manually generated by human coders.

### Statistical analysis

Observed percentage agreements and Cohen’s kappa coefficients were calculated using an open-source online statistical tool [26].

## Results

### Group characteristics

Table 1 outlines the total number of messages across each WhatsApp group based on the three supervisory categories: (i) Communication and information, (ii) Supportive environment and (iii) Quality Assurance.

**Table 1:** Breakdown of messages exchanged across the data sets according to supervisory category

	Training data set (*n* = 437)		Validation data set (*n* = 1242)	
	Combined human coder categorisation (*n* (%))	CHWsupervisor App categorisation (*n* (%))	Combined human coder categorisation (*n* (%))	CHWsupervisor App categorisation (*n* (%))
Communication and information messages	308 (70.5%)	286 (65.5%)	565 (45.5%)	498 (40.1%)
Supportive environment messages	77 (17.6%)	114 (26%)	581 (46.8%)	692 (55.7%)
Quality Assurance messages	52 (11.9%)	37 (8.5%)	96 (7.7%)	52 (4.2%)

A breakdown of messages exchanged across the datasets according to their supervisory category

### Human inter-coder reliability

#### Training test set

From the training test set, human inter-coder observed percentage agreement for the primary supervisory category of messages was 88% and the Cohen’s kappa coefficient was 0.70 (SE 0.04; CI 0.63–0.78).

#### Validation test set

From the validation test set, human inter-coder observed percentage agreement for the primary supervisory category of messages was 95% and the Cohen’s kappa coefficient was 0.91 (SE 0.09; CI 0.90–0.94).

### CHWsupervisor web app predictive reliability

#### Training test set

From the training test set, the observed percentage agreement for the primary supervisory category between the human coders was 78% and the Cohen’s kappa coefficient was 0.56 (SE 0.04; CI 0.49–0.64).

#### Validation test set

From the validation test set, the observed percentage agreement for the primary supervisory category between the human coders was 73% and the Cohen’s kappa coefficient was 0.51 (S.E: 0.02; CI: 0.47–0.56).

A summary of all inter-observer agreement statistics for both the training and validation data sets can be found in Table 2.

**Table 2:** Summary table of inter-observer agreement statistics for the training (Uganda) and validation (Kenya) data sets

	Training data set (Uganda)	Validation data set (Kenya)
Human coder agreement [observed % agreement; Cohen’s Kappa value (SE; CI)]	88%; 0.7 (SE 0.04; CI 0.63–0.78)	95%; 0.91 (0.091; 0.895–0.937)
CHWsupervisor web app predication accuracy [observed % agreement; Cohen’s Kappa value (SE; CI)]	78%; 0.56 (0.04; 0.49–0.64)	73%; 0.51 (SE 0.021; CI 0.473–0.556)

A summary of observed percentage agreements and Cohen’s Kappa values for inter-human-coder agreements and CHWsupervisor web app prediction accuracy across the training and validation test sets.

Please refer to the Additional files for the confusion matrices for the training and validation data sets (see Additional file 4).

### Time taken to code messages

Once the CHWsupervisor web app had been developed it took 46 min and 50 s unattended to code the full validation data set (1242 messages), in comparison to 12–14 h taken by each of the human coders. This was using a 4-year-old laptop with an Intel Core i7-7500U processor @2.70 GHz and 16 GB of installed memory.

## Discussion

The findings from our study suggest that human inter-coder reliability for the primary supervisory category of digital messages is superior to that of a machine learning web app (CHWsupervisor). This was demonstrated by the fact that human inter-coder agreement across the training and validation datasets was ‘substantial’ to ‘almost perfect’, as suggested by observed percentage agreements of 88–95% and Cohen’s kappa values of 0.7–0.91. In comparison to the human coders, the predictive accuracy of the CHWsupervisor web app was ‘moderate’, suggested by observed percentage agreements of 73–78% and Cohen’s kappa values of 0.51–0.56.

This work builds upon earlier studies which have attempted to better understand mHealth-facilitated CHW supervision, including those by Henry et al. and Pimmer et al. [13, 14]. In both of these studies, the research teams manually coded WhatsApp supervisory exchanges between CHWs in Kenya and Malawi; however, no details were provided as to how long this process took, nor the agreement between coders. Similar to our study, Henry et al. coded the messages based on their supervisory category using the same coding framework and found that the majority of supervisory exchanges fell under the categories of ‘Communication and Information’ (33.4%) and ‘Supportive Environment’ (64.7%), with only 19% of exchanges related to ‘Quality Assurance’ [13].

Having a better understanding of the nature of supervisory exchanges at an individual level could help identify those members of the supervisory network who take on ‘positive’ roles within the group (i.e. those sending a high proportion of messages tagged as ‘Supportive Environment’), as well as identify those CHWs who may appear to be less engaged. Having these insights could allow for tailored and personalised supervisory feedback, which has been documented as one way to increase CHW productivity in the existing literature [27]. Similarly, if those at an organisational level (e.g. programme managers and supervisors) had an overview of the nature of digital supervisory exchanges, it could allow for greater insights regarding the focus of supervisory interactions and individual actor involvement. Analysis and feedback of supervisory exchanges is something which has been suggested as important in the wider literature on ‘good supportive supervision’ [28], but is a current gap in the existing literature on supervision specific to CHWs. Given the findings of our study, we suggest that a machine learning approach could be one potential way to help achieve this at scale; however, it requires refinement before it is widely adopted.

One of the major challenges of our study, which is perhaps one of the reasons why we observed lower predictive scores of the CHWsupervisor app compared to human-to-human ratings, is the complex nature of supervisory exchanges; these are nuanced, contextually situated, and often require an understanding of the actors involved and the nature of the dynamic dialogue. Unlike prior uses of machine learning in biomedical science in which there is a binary outcome (for example, the presence or non-presence of ocular pathology) [29], supervisory exchanges can contain multiple layers of interactions embedded within one message, can occur between multiple different actors within a dynamic space, and can be more open to individual interpretation.

One of the other limitations of the present analysis is that we focussed on the primary supervisory category of the message; however, there was a subset of messages which contained multiple different types of supervisory interactions. The CHWsupervisor web app did not have the ability to distinguish between first-, second- and third-order supervisory categories within one message, but rather produced a ‘confidence percentage’ as to the likelihood of the message being in one of the three broad supervisory categories. Future iterations of the web app could therefore be developed to attempt to distinguish between high-, mid- and lower-order categories within complex supervisory exchanges.

Similarly, subtle linguistic nuances were not detected by the CHWsupervisor web app. For example, some messages were coded by the app as ‘Communication and Information’ since they appeared to be logistical messages asking the CHWs if they needed any help; however, the human coders inferred the tone of some of these messages as ones which were aimed at creating a ‘Supportive Environment’ given the encouraging nature.

Next, CHWsupervisor was only trained to code messages across three broad categories of supervision. This is likely to be useful to CHW programme managers and supervisors who wish to understand the general nature of supervision that is occurring. It can also allow supervisors to focus on areas they are particularly interested in. For example, highlighting and analysing messages related to ‘Quality Assurance’ may allow supervisors to understand the nature of messages related to public health messages or health promotion, and allow them to sub-analyse these for health mis-information (which has been documented as a concern regarding the use of instant-messaging platforms, such as WhatsApp, amongst healthcare workers) [30]. However, the analysis did not extend to a more detailed sub-analysis of supervisory exchanges. For example, messages related to ‘Quality Assurance’ could be further sub-categorised into areas related to follow-up, household visits, health education and information, or referrals. Future work on subsequent models of the app could aim to capture this level of detail; however, the predictive accuracy at this level of sub-analysis may prove challenging given that amongst just three broad supervisory categories predictive accuracy dropped from ‘substantial’ to ‘moderate’ when comparing human-generated codes against the CHWsupervisor web app-generated codes.

Regarding the transferability of the App, CHWsupervisor was developed and validated on messages exchanged between cohorts of CHWs in East Africa. Whilst we view it as a strength of our work that we validated the app on a second set of independent data from Kenya, it is possible that the nature of message exchanges between other CHWs from different cultures and geographic regions may affect the reliability of the app to accurately predict supervisory exchanges between CHWs from different regions of the world. Further validation of the app using alternative datasets should therefore be explored. Another technical limitation with the web app in its current form is that it is only able to detect and code text-based messages. This is a significant limitation given that almost half (49.7%) of messages exchanged by the CHWs in the data set from Uganda were voice messages. Furthermore, although the app can only process messages in English, the underlying technology is available in 16 different languages. Future work will therefore focus on automated transcription of voice-notes to text messages. It is also important to note that although WhatsApp was the instant-messaging platform used to develop and validate CHWsupervisor, all that is required to use the app is a spreadsheet containing the original messages. It should also be noted that the average message from the training data set (Uganda) contained 3.7 sentences (264 characters), while the validation data set (Kenya) was only 1.4 (78). This could have contributed to the moderate performance in the training data set in comparison to the validation data set and future work could explore predictive accuracy using message length and complexity as a variable. A further limitation with regards to the datasets used in this study was the relatively high ratio of CHW supervisors to CHWs, which is not always the reality on the ground. In both studies, remote supervision using mobile technologies did not pre-exist so it allowed the research team to establish remote supervision using WhatsApp with relatively small numbers of CHWs. It is therefore important to assess and evaluate this approach at scale in other programmes where supervisee to supervisor ratios are much higher. Likewise, only one member of the research team sorted and cleaned the dataset which may have led to potential bias. Finally, given the CHWsupervisor web app was able to code the validation data set of 1242 messages 16 times faster than the fastest human coder (45 min 50 s vs. 12 h), such an approach does hold some promise if it were to be optimised given the speed at which large data sets could be analysed. Given the growth in digitally assisted CHW supervision, we therefore suggest further refinement and testing of the app is warranted.

## Conclusions

Despite claims that machine learning could “transform global health care in a myriad of ways” [31], little empirical work has been conducted to explore the potential application of such strategies to CHW supervision—an important but underexplored component of health systems strengthening in LMICs. This current study is one of the first of its kind to apply a machine learning approach to the analysis of digital supervisory messages between CHWs. Our open access machine learning web app was able to predict the nature of supervisory exchanges with ‘moderate agreement’ when compared to human-coders. Although such an approach could help those responsible for moderating and facilitating CHW supervision to better understand the general nature of supervision occurring between CHWs at scale, our study was not without its limitations, of which there were several. These included challenges with the app being able to accurately predict the nuanced nature of more complex and lengthy message exchanges, and potential issues with transferability to other contexts. As a result, we caution viewing machine learning approaches to supervisory analysis at scale as a panacea, but highlight the continued need for human expertise given the nuanced complexities of supervisory exchanges.

## Supplementary Information

**Additional file 1**: Data cleaning process for test and validation data sets.

**Additional file 2: **Links to libraries underlying the CHWsupervisor web app.

**Additional file 3: **Additional CHWsupervisor web app development details.

**Additional file 4: **Confusion matrices.

## Data Availability

The source codes used in this study are available online (see additional material for further details). The datasets generated and/or analysed during the current study are not publicly available due to the sensitive nature of certain topics discussed and for matters relating to confidentiality. Sharing of the data from the private WhatsApp groups was not granted during the ethical approval process for this study. They are therefore not available to be shared.

## Abbreviations

AI: Artificial intelligence
CHW: Community Health Worker
LMIC: Low- and middle-income country
mHealth: Mobile Health
WHO: World Health Organization
