# Supplementary material for: Analysing 3429 digital supervisory interactions between Community Health Workers in Uganda and Kenya: the development, testing and validation of an open access predictive machine learning web app
Source: Hum Resour Health. 2022 Mar 16;20:6. doi: 10.1186/s12960-021-00699-5 (PMC8925202; doi:10.1186/s12960-021-00699-5)
Supplement: Supplementary file 1 — Additional file 1: Data cleaning process for test and validation data sets. [file 12960_2021_699_MOESM1_ESM.docx]

### Additional File 1. Data cleaning process for test and validation data sets

All non-text messages, nonsensical messages, non-supervisory messages and all duplicate messages were removed prior to analysis. The summary breakdown of these removed messages can be found below in Supplementary Table 1.

**Supplementary Table 1. Breakdown of messages removed from the test and validation data sets prior to final analysis.**

| **Message category** | **Test data set** | **Validation data set** |
| --- | --- | --- |
| Media only messages (e.g. photos/videos) | 231 | 447 |
| Blank messages/voice notes | 32 | 13 |
| Political or religious messages | 31 | 17 |
| Nonsensical messages | 24 | 108 |
| Links to local news | 11 | 0 |
| Automated system notifications/alerts | 0 | 51 |
| Duplicate or repeat message | 1 | 7 |
| **Total** | **330** | **643** |
