# Supplementary material for: Analysing 3429 digital supervisory interactions between Community Health Workers in Uganda and Kenya: the development, testing and validation of an open access predictive machine learning web app
Source: Hum Resour Health. 2022 Mar 16;20:6. doi: 10.1186/s12960-021-00699-5 (PMC8925202; doi:10.1186/s12960-021-00699-5)
Supplement: Supplementary file 2 — Additional file 2: Links to libraries underlying the CHWsupervisor web app. [file 12960_2021_699_MOESM2_ESM.docx]

**Additional File 2. Links to libraries underlying the CHWsupervisor web app**

The libraries underlying the web app are available at: <https://github.com/ecraft2learn/ai> and <https://github.com/tensorflow/tfjs>.
