# Supplementary material for: Analysing 3429 digital supervisory interactions between Community Health Workers in Uganda and Kenya: the development, testing and validation of an open access predictive machine learning web app
Source: Hum Resour Health. 2022 Mar 16;20:6. doi: 10.1186/s12960-021-00699-5 (PMC8925202; doi:10.1186/s12960-021-00699-5)
Supplement: Supplementary file 3 — Additional file 3: Additional CHWsupervisor web app development details. [file 12960_2021_699_MOESM3_ESM.docx]

**Additional File 3. Additional CHWsupervisor web app development details**

### The first step in creating the CHWsupervisor involved repeatedly randomly splitting the WhatsApp Group B dataset of 1078 messages into 60% for training (n=647), 20% for fine-tuning (n=215), and 20% for testing (n=215).

### The model’s labelling matched one of the ratings 88% of the time and matched the primary category 74% of the time, the secondary category 11% (if one was given) and 1% match of the third category. 56% of the messages had been rated by humans with only one category, 41% had two, and 3% had all three. The model’s confidence was below threshold for 1% of the messages and were not labelled. All the other tests left less than 0.5% of their test messages unlabelled.

### We repeated this for Group A. Messages in this dataset had one category 51% of the time, 47% two, and 2% three. The model matched the ratings for 90% of the messages, where 81% matched the primary category, 9% the secondary category, and 1% the tertiary category.

### A model trained on the combination of the two datasets produced labels that matched one of the rated categories 87% of the time, where 78% matched the first category, 8% the second and 1% the third. This is the model that is used when a user chooses to label their messages without first training using their own data.

### When we trained models on all of Group A and then tested it on Group B, the system was correct 81% where 68% matched the primary category, 11% the secondary, and 2% the tertiary. When trained on Group B and tested on Group A it was correct 89% where the primary category match was 74%, the secondary 13% and the tertiary 1%.

### Random guessing averaged 33% correct for the primary category and 50% for any.

### In order to evaluate how well the app might perform with less data, we repeated the trained on A then tested on B and also trained on B then tested on A. We evaluated models trained with ½, ¼, and 1/8^th^ of all the data. Group A’s accuracy dropped to 72%, 81%, and 75% respectively. When inspecting the training with ½ the data we saw that the accuracy was still improving when it reached the limit of 2000 training steps. Increasing the limit to 5000 brought it up to 81%. Group B’s accuracy dropped to 72%, 69%, and 63%. Increasing the epoch limit to 5000 only improved the ¼ data training to 72% from 69%.

It is common practice in machine learning to adjust the parameters that define the model's architecture and training settings. After a period of fine-tuning we found that the following hyper-parameters performed well:

•         The model consists of a hidden layer of 64 artificial neurons fully connected to the input encoding and to the next hidden layer of 16 neurons (N.B. deeper and wider models did not perform better)

•         On each training step model parameters were adjusted by the product of the gradient and values between 0.0007 and 0.005

•         To avoid overfitting on every training step 0.5 of the model parameters are randomly selected and set to zero in a technique known as dropout

•         Training stopped after either 2000 steps or after many steps with no improvement in accuracy measured against a held-out validation dataset

### The CHWsupervisor web-app also produces secondary and tertiary labels when confidence levels are high. We have not evaluated how accurate these are.

**Challenges due to the lack of balance between the categories**

As can be seen in Supplementary Table 2, while the frequency of “Communication and Information” and “Supportive Environment” are roughly equal “Quality Assurance” ranges from 5 to 9% of the total. This causes problems for the machine learning app. Trained models can have high accuracy even if they never predict “Quality Assurance”. For example, a model trained on 60% of the combined datasets has the following confusion matrix:

**Supplementary Table 2. Unbalanced combined datasets**

|  | **True Communication**  **and Information** | **True Supportive Environment** | **True Quality Assurance** |
| --- | --- | --- | --- |
| **Predicted Communication and Information** | **266 (60.7%)** | 38 (8.7%) | 25 (5.7%) |
| **Predicted Supportive Environment** | 18 (4.1%) | **79 (18%)** | 1 (0.2%) |
| **Predicted Quality Assurance** | 6 (1.4%) | 0 (0%) | **5 (1.1%)** |

Without balancing the training data, it predicted “Quality Assurance” only 11 times (and only 5 of those were correct) while there were 31 occurrences in the test set. In addition to prediction accuracy the models need to be evaluated with regard to recall and precision. In machine learning it is common in similar situations to evaluate models with F1 scores. One solution is to train the models with a balanced dataset. Doing so with the combined dataset leaves 200 message for each category. When we split this dataset into 60% for training, 20% for tuning and validation, and 20% for testing we encountered a significant drop in accuracy despite increases in F1 from 54 to 56% with the unbalanced dataset to 62% to 75% (the wide range of results we assume is due to the stochasticity due to the small training dataset). Accuracy dropped from 90% to 80%. Attempts to address this problem by biasing the loss function used in training to weigh more heavily the rare categories produced an even greater drop in accuracy. See Supplementary Table 3.

**Supplementary Table 3. Balanced combined datasets**

|  | **True Communication**  **and Information** | **True Supportive**  **Environment** | **True Quality Assurance** |
| --- | --- | --- | --- |
| **Predicted Communication and Information** | **31 (26.5%)** | 8 (6.8%) | 6 (5.1%) |
| **Predicted Supportive Environment** | 5 (4.3%) | **31 (26.5%)** | 2 (1.7%) |
| **Predicted Quality Assurance** | 7 (6%) | 2 (1.7%) | **25 (21.4%)** |

The consequences of balancing the data when training with Group A (B) to label Group B (A) the accuracy and F1 scores dropped to below acceptable levels.

One solution we did not pursue involves handcrafting hundreds of sample Quality Assurance messages for use in training.

The best solution we were able to implement was to duplicate some training messages to partially balance the data. When trained with 40% Communication & Information, 33% Supportive Environment, and 27% for Quality Control we were able to match the primary category 78%, secondary category 8% and tertiary category 1%. The average F1 score was 70%.

We also investigated how well the app would work with less data (leaving it unbalanced). After training with Group A (B) with ½, ¼, and 1/8^th^ of the data, we investigated the accuracy drop when then applied to labelling the other group. We found that accuracy dropped between 14 and 27% when training with the reduced Group A data while only 7 to 14% for the Group B data.
