# Supplementary material for: Analysing 3429 digital supervisory interactions between Community Health Workers in Uganda and Kenya: the development, testing and validation of an open access predictive machine learning web app
Source: Hum Resour Health. 2022 Mar 16;20:6. doi: 10.1186/s12960-021-00699-5 (PMC8925202; doi:10.1186/s12960-021-00699-5)
Supplement: Supplementary file 4 — Additional file 4: Confusion matrices. [file 12960_2021_699_MOESM4_ESM.docx]

### Additional File 4. Confusion Matrices

**Supplementary Table 4. Confusion matrices for development test set messages coded between human coder 1 and human coder 2 (n=437).**

|  | **Human coder 2 –‘Communication and Information’ (n,(%))** | **Human coder 2 –‘Supportive Environment’ (n,(%))** | **Human coder 2 –‘Quality Assurance’ (n,(%))** |
| --- | --- | --- | --- |
| **Human coder 1 – ‘Communication and Information’ (n,(%))** | **301 (68.9%)** | 3 (0.7%) | 4 (0.9%) |
| **Human coder 1 – ‘Supportive Environment’ (n,(%))** | 15 (3.4%) | **62 (14.2%)** | 0 |
| **Human coder 1 – ‘Quality Assurance’ (n,(%))** | 30 (6.9%) | 1 (0.2%) | **21 (4.8%)** |

**Supplementary Table 5. Confusion matrices for validation test set messages coded between human coder 1 and human coder 2 (n=1242).**

|  | **Human coder 2 –‘Communication and Information’ (n,(%))** | **Human coder 2 –‘Supportive Environment’ (n,(%))** | **Human coder 2 –‘Quality Assurance’ (n,(%))** |
| --- | --- | --- | --- |
| **Human coder 1 – ‘Communication and Information’ (n,(%))** | **537 (43.2%)** | 22 (1.8%) | 6 (0.5%) |
| **Human coder 1 – ‘Supportive Environment’ (n,(%))** | 20 (1.6%) | **561 (45.2%)** | 0 |
| **Human coder 1 – ‘Quality Assurance’ (n,(%))** | 10 (0.8%) | 1 (0.1%) | **85 (6.8%)** |

**Supplementary Table 6. Confusion matrices for development test set messages coded between CHWsupervisor web app and human coders (n=437).**

|  | **Combined human coder – ‘Communication and Information’ (n,(%))** | **Combined human coder – ‘Supportive Environment’ (n,(%))** | **Combined human coder – ‘Quality Assurance’ (n,(%))** |
| --- | --- | --- | --- |
| **Machine Learning Predicted Communication and Information (n,(%))** | **254 (58.1%)** | 8 (1.8%) | 24 (5.5%) |
| **Machine Learning Predicted Supportive Environment (n,(%))** | 39 (8.9%) | **69 (15.8%)** | 6 (1.4%) |
| **Machine Learning Predicted Quality Assurance (n,(%))** | 15 (3.4%) | 0 | **22 (5%)** |

**Supplementary Table 7. Confusion matrices for validation test set messages coded between CHWsupervisor web app and human coders (n=1242).**

|  | **Combined human coder -‘Communication and Information’ (n,(%))** | **Combined human coder – ‘Supportive Environment’ (n,(%))** | **Combined human coder – ‘Quality Assurance’ (n,(%))** |
| --- | --- | --- | --- |
| **Machine Learning Predicted Communication and Information (n,(%))** | **372 (30%)** | 56 (4.5%) | 70 (5.6%) |
| **Machine Learning Predicted Supportive Environment (n,(%))** | 158 (12.7%) | **522 (42%)** | 12 (1%) |
| **Machine Learning Predicted Quality Assurance (n,(%))** | 35 (2.8%) | 3 (0.2%) | **14 (1.1%)** |
